# Quality Improvement Project (QIP) to Review Treatment Forms (T2B/T3B) for NHS Greater Glasgow and Clyde Community Mental Health Teams (CMHT) Patients

**DOI:** 10.1192/bjo.2026.11374

**Published:** 2026-06-30

**Authors:** Mai Elsawaf

**Affiliations:** NHS Greater Glasgow and Clyde, Glasgow, United Kingdom

## Abstract

**Aims:**

To assess whether information recorded on T2B and T3B treatment forms accurately corresponds to medications prescribed to patients by Community Mental Health Teams (CMHT) or General Practitioners (GPs). The hypothesis was that discrepancies may exist due to the absence of inpatient safeguards in community settings.

**Methods:**

Under the Mental Health (Care and Treatment) (Scotland) Act 2003, patients on compulsory treatment for more than two months require T2B or T3B forms. T2B for consented treatment and T3B for treatment approved by a Designated Medical Practitioner (DMP). These forms protect patient rights and must be completed accurately. Guidance is provided by the Mental Welfare Commission through publications such as Consent to Treatment (2017) and Medical Treatment under Part 16 (2021), with an updated consent guide published in October 2025 (postdating this audit).

T2B forms should specify exact medications while T3B may list broader drug classes. Inpatient settings have safeguards like electronic records alerts, dispensing checks, and MDT oversight. These are absent in community care, where CMHTs request medication changes via GPs and do not dispense most medications directly. If patients are reviewed by clinicians other than their Responsible Medical Officer (RMO), there is a risk of unauthorized treatment.

**Methods:**

The audit reviewed all patients on T2B and T3B forms under NHSGGC Adult CMHTs (Renfrewshire, Inverclyde, South Glasgow, North East Glasgow, and North West Glasgow HSCPs) with Caldicott approval. A checklist assessed whether forms were in date, uploaded to EMIS (electronic records), included consent (for T2B), referenced in clinicletters, and matched ECS (GP Emergency Care Summary) records.

Medical records provided listing of patients on Compulsory Treatment Orders (CTO) and treatment forms. Separate audits for each site were conducted by core trainees and reviewed by Dr Mai Elsawaf who combined a report. Data access occurred between June and October 2025, though timing varied between teams, a noted limitation.

**Results:**

A total of 558 patients were included (491 General Adult, 36 Forensic, 8 Learning Disability, 23 Older Adults). Most forms were for CCTO (Community Compulsory Treatment Order) patients, aligning with the audit’s scope. T3B forms were more common than T2B, reflecting treatment as a key reason for CCTO. Compliance was high, with 99.8% of forms and consent uploaded to EMIS.

However, only 85% of clinic letters mentioned treatment orders, and just 14% referenced treatment forms, limiting information sharing with GPs and emergency teams. Medication alignment was strong (90%), with discrepancies due to additional psychotropics. ECS correspondence was lower (80%), with missing or incorrect entries posing risks, especially for clozapine, which was absent in two cases. Other mismatches were due to dose adjustments not reflected in forms. Best practice includes reissuing updated forms or using dose ranges and alternative plans on T3B forms.

**Conclusion:**

The audit showed 90% correspondence between treatment forms and prescribed medications, with main discrepancies from additional medications and poor documentation in clinic letters. Actions taken included contacting relevant teams with discrepancies and changing the format of the clinic letters to include information on treatment forms. Recommendations include re-circulating guidance, adding EMIS alerts, considering inclusion of treatment form details in clinic letters, and re-auditing in 12 months.

